# Immunogenicity and protective efficacy against *Treponema pallidum* in New Zealand rabbits immunized with plasmid DNA encoding flagellin

**DOI:** 10.1038/s41426-018-0176-0

**Published:** 2018-11-07

**Authors:** Kang Zheng, Man Xu, Yongjian Xiao, Haodang Luo, Yafeng Xie, Jian Yu, Manyi Tan, Yumeng Li, Feijun Zhao, Tiebing Zeng, Yimou Wu

**Affiliations:** 1 Institute of Pathogenic Biology, Medical College, University of South China, Hunan Province Cooperative Innovation Center for Molecular Target New Drug Study, Hengyang, 421001 China; 2 Clinical Laboratory, The Second Affiliated Hospital of University of South China, Hengyang, 421001 China; 3 Department of Experimental Zoology, Medical College, University of South China, Hengyang, 421001 China

## Abstract

Plasmid DNA encoding flagellin FlaB3 was used as a vaccination candidate for the evaluation of immunogenicity and protection against *Treponema pallidum* subsp. *pallidum* dissemination. First, intramuscular injection of the flagellin encoded by the plasmid DNA into New Zealand rabbits elicited both humoral and cellular immune responses. Total IgG production increased in response to flagellin. In addition, serum IFN-γ secretion and CD8+ cells were substantially greater in the rabbits immunized with the plasmid encoding flagellin FlaB3 than those in the rabbits immunized with recombinant flagellin. The flagellin encoded by the plasmid DNA induced significant upregulation of serum IL-6 and IL-8 compared to that of the control rabbits. Subsequently, intradermal challenge of the vaccinated New Zealand rabbits with 1 × 10^7^*T. pallidum* resulted in a significant reduction of the bacterial organ burden in the blood, liver, spleen, and testicles in the flagellin plasmid DNA-vaccinated rabbits. Furthermore, the histopathological analysis demonstrated that the rabbits immunized with the plasmid DNA-encoded flagellin (FlaB3) showed better immune protection. These findings provide evidence that plasmid DNA-encoded flagellin (FlaB3) may be useful as a potential immunization route for future development of a vaccine to inhibit *T. pallidum* dissemination in related animals.

## Introduction

As a sexually transmitted infection, syphilis typically occurs as a result of infection with *Treponema pallidum* subsp. *pallidum* (*T. pallidum*) and is most commonly reported in low- and middle-income countries^1,2^. Human infection with *T. pallidum* usually occurs via mucous membranes or injured skin^3,4^. The reported per annum estimated frequency of infectious syphilis is 36 million cases and more than 11 million new infections^5^. However, the number of cases reported is believed by some to be a significant underestimate. Thus, this disease represents an important global public health burden^6^.

To date, developed vaccines against *T. pallidum* have included an inactivated vaccine, live attenuated vaccines, recombinant protein vaccines and DNA vaccines^7–9^. γ-Irradiated *T. pallidum*, which is an attenuated syphilis spirochete, has been used as an attenuated vaccine to induce a protective antibody response against *T. pallidum* in vivo, although the relative efficacy of this vaccine has not been well defined in animal trials^8^. The *T. pallidum* laminin-binding adhesin Tp0751 has been reported to induce high titers of specific antibodies and results in delayed lesion development, inhibition of *T. pallidum* dissemination and increased cellular infiltration at lesion sites^10^. In another example, sera from immunized rabbits were reported to have anti-endoflagellar antibody titers that were fivefold greater than the serum titers from infected immune rabbits and thus provided some level of passive protection^11,12^. Typically, the use of recombinant antigens as vaccination candidates induces powerful humoral antibodies but may not induce some form of cell-mediated immune response^13^. However, sera from *T. pallidum*-infected patients display a superiority of IL-2, IFN-γ, and TNF-α (Th1 or Th1-like cytokines) production and low IL-4 and IL-5 (Th2 or Th2-like cytokines) levels^14^. Due to the presence of protective humoral antibodies, *T. pallidum* is able to invade the skin or genital mucosa, penetrate the tissue barrier, and gain rapid entry to the bloodstream^3^. If not completely eliminated from an infected individual, *T. pallidum* can cause diverse clinical manifestations, including skin rashes, meningitis, ocular disease, and cardiovascular and neurological complications^6,15^. More seriously, *T. pallidum* can permeate both the placental and blood–brain barriers and increases the risk of acquiring and transmitting human immunodeficiency virus (HIV)^15–17^. Therefore, effective syphilis vaccine development needs to target treponemes within the vasculature to inhibit *T. pallidum* seeding into a wide variety of tissues and organs^9^.

Bacterial flagellin has emerged as a potent immunostimulator that is capable of activating the MyD88-dependent signaling pathway and NF-κB-mediated production of proinflammatory cytokines and nitric oxide (NO) in vivo, which contribute to immediate clearance of pathogens from the host^18–20^. Our previous study showed that *T. pallidum* flagellins upregulated IL-6 and IL-8 via the TLR5 and MAPK/NF-ƙB signaling pathways in THP-1 cells^21^. In another study, *T. pallidum* flagellin (FlaB1, FlaB2, or FlaB3) stimulation augmented the production of MMPs (matrix metalloproteinases) in HaCaT cells via the MyD88-dependent TLR5 signaling pathway^22^. Furthermore, the filament core protein FlaB3 is homologous to the N-terminal and C-terminal regions of the flagellins of *Salmonella enterica* and *Escherichia coli*^23,24^. With these encouraging observations, flagellin has been targeted as an immunogenic agent for the development of a vaccine to inhibit *T. pallidum* dissemination.

DNA vaccines are capable of inducing long-term antibody responses, which are the principal agents of immune protection against most viruses and bacteria^25^. Nucleic acid vaccines against hepatitis B virus, influenza virus, and *Mycobacterium tuberculosis* are in the preclinical stage of evaluation or have entered clinical trials^26–28^. Hence, this study was designed to evaluate the relative immunogenicity and inhibition of *T. pallidum* dissemination in New Zealand rabbits by intramuscular injection of a plasmid DNA vaccine carrying the *T. pallidum* flagellin *flaB3* gene.

## Results

### Antibody response

**Fig. 1:**
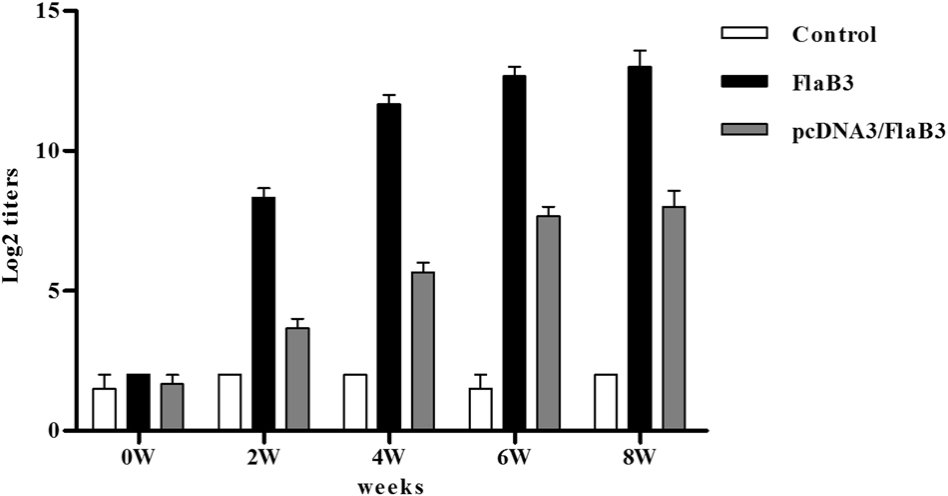
Induction of the anti-flagellin IgG response. New Zealand rabbits were immunized with 250 μg of the pcDNA3/FlaB3 plasmid, 150 μg of pcDNA3 as a negative control or 150 μg of recombinant flagellin as a positive control. The serum IgG levels were analyzed by ELISA. The means and standard derivations for the antibody levels were calculated from three rabbits

pcDNA3/FlaB3 was transfected into HeLa cells. After 48 h, the total proteins from these transfected cells reacted with anti-flagellin sera by western blotting and gave a specific immunoreactive band of 34 kDa (Fig. S1). The results confirm that the plasmid construct pcDNA3/FlaB3 is intact and functional in eukaryotic cells. To determine whether pcDNA3/FlaB3 was able to induce an antibody response in rabbits, New Zealand rabbits were injected with pcDNA3/FlaB3 intramuscularly, and an ELISA was performed to measure the level of flagellin-specific antibodies in their sera. A specific antibody was produced in the pcDNA3/FlaB3 group at week 2, and then the antibody titers against flagellin gradually increased (Fig. 1). This result was in contrast to the positive control rabbits injected with 150 µg of recombinant flagellin (FlaB3), which exhibited a prominent antibody response in their sera (Fig. 1). The results clearly show that intramuscular injection of pcDNA3/FlaB3 into New Zealand rabbits generates a specific antibody response.

### Cellular immune response

**Fig. 2:**
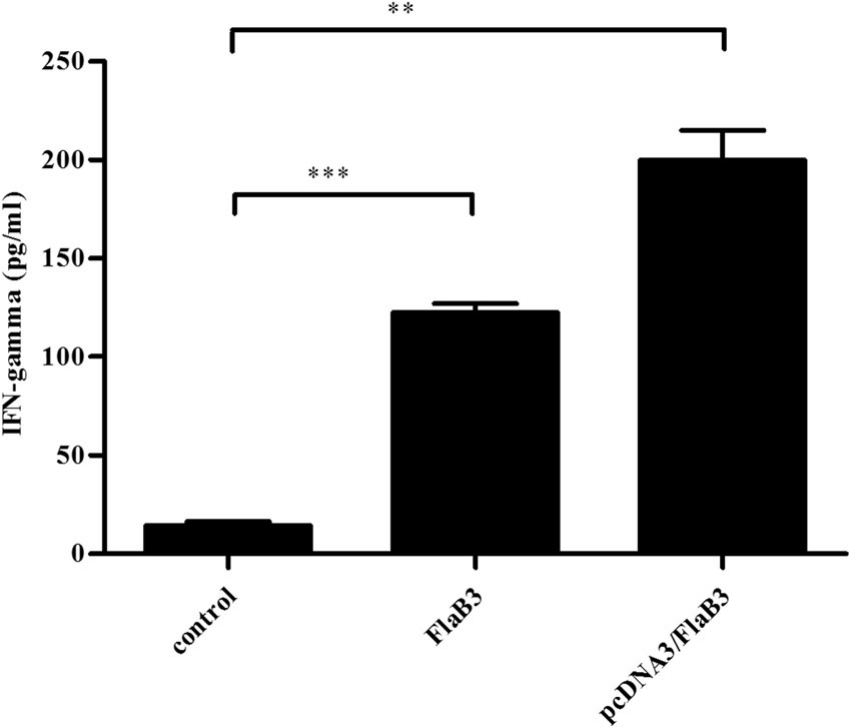
Production of cytokines in the sera of the pcDNA3-, recombinant FlaB3-, and pcDNA3/FlaB3-immunized rabbits. Sera collected from immunized and nonimmunized rabbits were evaluated with an IFN-γ ELISA kit according to the manufacturer’s protocols. The means ± SDs are the results from three individual rabbit sera in three independent experiments (**P < 0.01, ***P < 0.001)

As confirmed previously, IFN-γ is the most important Th1-related cytokine involved in early defense against *T. pallidum* infection^29–33^. T cells of both the CD4 and CD8 lineages are needed for protective anti-*T. pallidum* immunity^32^. Hence, we assessed the serum levels of IFN-γ and CD8+ cells in the pcDNA3/FlaB3-immunized rabbits (Fig. 2, S2). The upregulation of IFN-γ and CD8+ cell production was significantly increased in the rabbits immunized with pcDNA3/FlaB3 compared with that of the rabbits immunized with either recombinant flagellin or vaccinated with the vector DNA alone.

### Cytokine response

To test whether recombinant flagellin and plasmid DNA-encoded flagellin could increase systemic IL-6 and IL-8 levels in vivo, the IL-6 and IL-8 concentrations in rabbit sera were determined using specific ELISA assays. Sera from rabbits immunized with either pcDNA3/FlaB3 or recombinant flagellin contained higher IL-6 and IL-8 levels than that of rabbits vaccinated with vector DNA alone (Fig. S3).

### Intramuscular vaccination with pcDNA3/FlaB3 attenuates lesion development

**Fig. 3:**
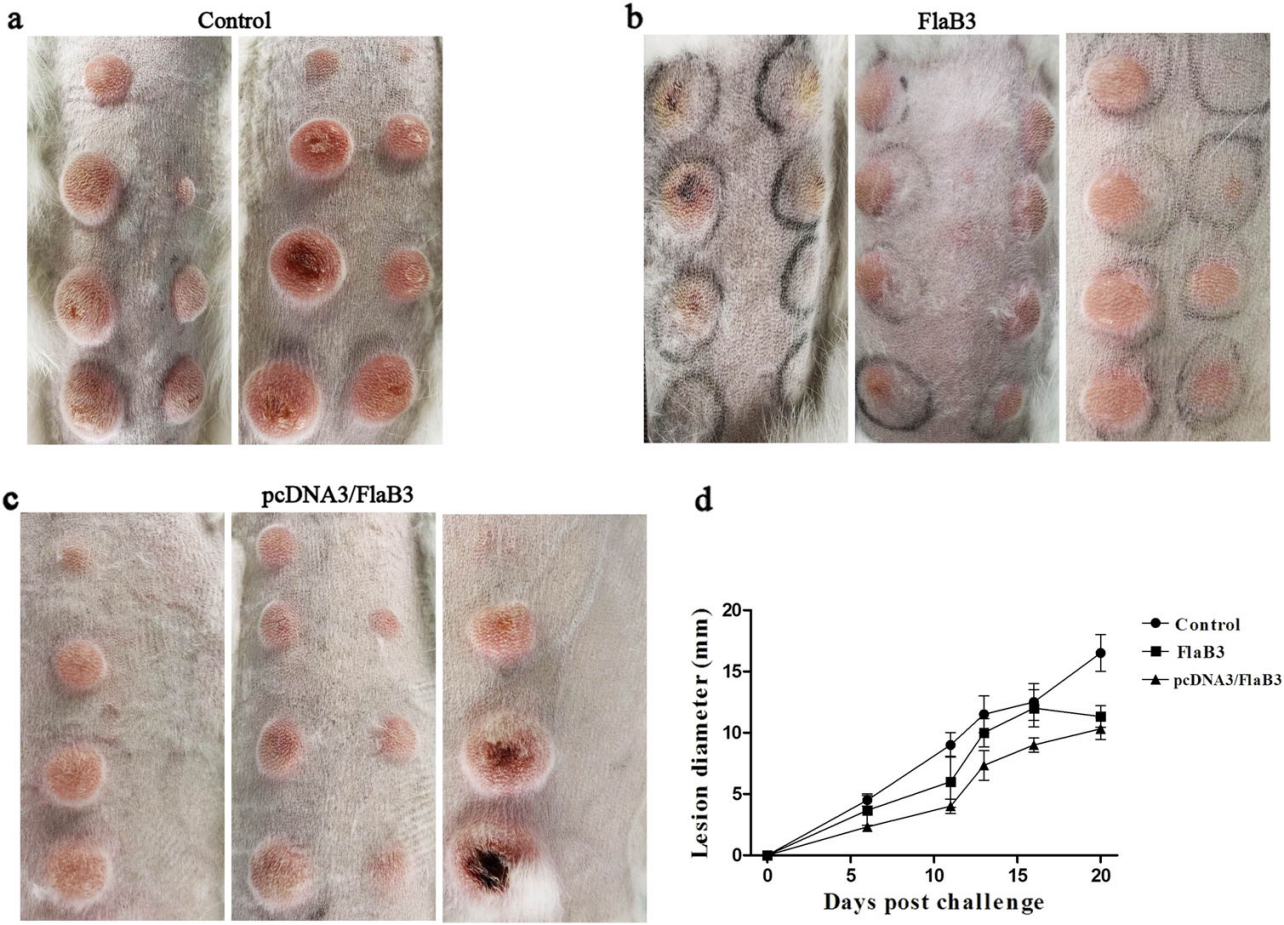
Immunization with pcDNA3/FlaB3 attenuates lesion development. The pcDNA3/FlaB3-immunized rabbits had fewer ulcerative lesions than the unimmunized controls and the lesion diameter measured in the control animals was larger than that in immunized animals within 21 days postinfection (**a**, **b**, **c**). Eight rabbits were collected on day 21 post-intradermal challenge with the *T. pallidum* Nichols strain at eight locations on their backs. Skin lesions were observed and measured at the challenged sites every 3 days (**d**). The results are presented as the median ± 95% confidence interval between individual immunized or control animals. Significance was assessed using nonlinear regression and the extra sum of squares F-test (*P* < 0.05 (**d**))

**Fig. 4:**
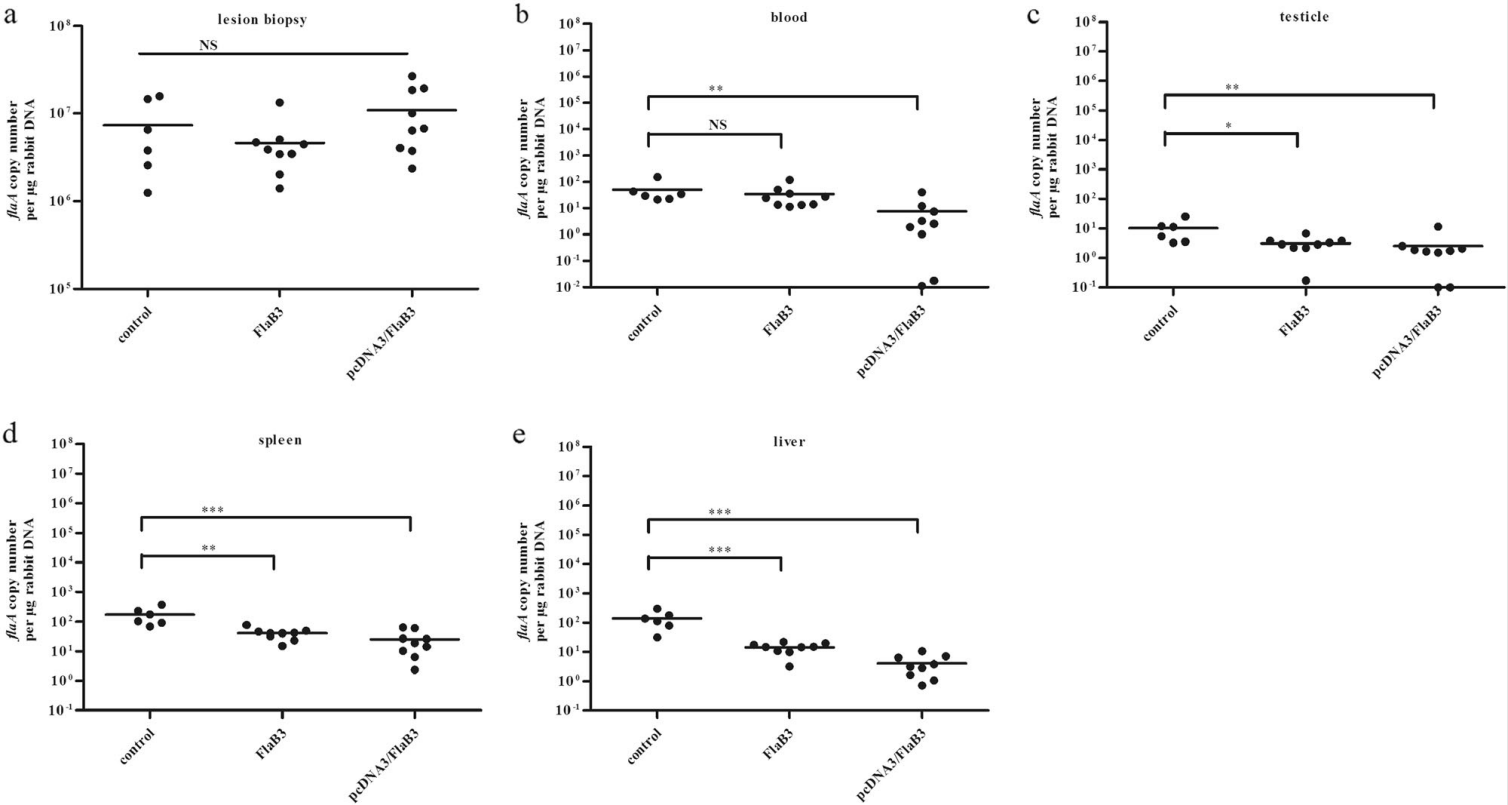
Immunization with pcDNA3/FlaB3 inhibits *T. pallidum* dissemination. The *T. pallidum* burden was evaluated in control animals (*N* = 2) and animals immunized with pcDNA3/FlaB3 (*N* = 3) or recombinant flagellin (*N* = 3) using quantitative real-time PCR to measure the *flaA* DNA concentrations in the lesion biopsies ((**a**); lesion biopsy) and disseminated infection sites ((**b**) blood, (**c**) testicles, (**d**) spleen, and (**e**) liver). The results were normalized to the rabbit gDNA concentrations using the Mann–Whitney test. The points correspond to three samples that were separately extracted from each animal. Horizontal lines represent mean values (*P < 0.05, **P < 0.01, ***P < 0.001, NS nonsignificant)

At 21 days following the final immunization, the immunized and control unimmunized animals were intradermally challenged with 1 × 10^7^*T. pallidum* subsp*. pallidum* (Nichols strain) per ml in 0.9% saline at each of eight sites on their shaved backs. Previous syphilis studies have found that monitoring chancre development provides a metric of vaccine efficacy^9^. Hence, skin lesions were observed and measured in the infected sites every 3 days. On day 14, lesions (rash and ulcerative) were present on all animals, but the control animals presented 100% lesion induration on days 6–8, whereas the animals immunized with pcDNA3/FlaB3 or FlaB3 matched the level of induration observed in the control animals until days 11–12 (Fig. 3a–c). Ulcerative lesions (ulceration but typically not purulent) in the control group were evident as early as day 14. However, the animals immunized with FlaB3 or pcDNA3/FlaB3 developed ulceration of the lesion on day 16, and the pcDNA3/FlaB3-immunized rabbits had fewer ulcerative lesions than the unimmunized controls (Fig. 3a–c). The lesion diameter measured in the control animals was larger than that in the immunized animals within 21 days postinfection (Fig. 3d). At 21 days post-*T. pallidum* infection, thirty lesions were investigated by darkfield microscopy. Rabbits immunized with pcDNA3/FlaB3 had significantly fewer lesions with detectable treponemes than the rabbits vaccinated with the recombinant protein (Table 1). These results indicate that the lesion ulceration, lesion diameter and treponemal burden are significantly decreased in the pcDNA3/FlaB3-immunized animals and that the former strategy is significantly superior to vaccination with the recombinant protein (Fig. 4).

**Table 1:** The numbers of *T. pallidum* in the lesions

Immunogen	*T. pallidum*Darkfield analysis (%)^a^	Lesion statusUlcerative lesions (%)^b^
pcDNA3/FlaB3	3/10 (30)	2/14 (14.29)
FlaB3	4/10 (40)	4/20 (20)
Control	8/10 (80)	6/15 (40)

^a^The denominator indicates the number of lesions examined by darkfield microscopy

^b^The denominator indicates the total number of lesions in the treatment group

### pcDNA3/FlaB3 immunization inhibits *T. pallidum* dissemination

To validate whether flagellin plasmid DNA immunization conferred protection against *T. pallidum* seeding into distal infection sites, quantitative PCR (qPCR) was used to assess the *T. pallidum* burdens in the primary lesion sites, blood, spleen, liver, and testicles on day 21 postinoculation. The results showed a slight trend toward increased *T. pallidum* concentrations at the primary lesion sites in the flagellin plasmid DNA-vaccinated rabbits compared to those of the recombinant protein-vaccinated rabbits (Fig. 4a). Somewhat contradictory to this observation, darkfield microscopy analysis of the primary lesion showed fewer treponemes in all immunized animals (Table 1). However, these results must be interpreted with caution, because residual DNA from nonintact spirochetes was detected by qPCR and contributed to the high treponemal levels. However, the treponemal burden in blood, spleen, liver, and testicles of the rabbits immunized with plasmid DNA was still significantly lower than that of the rabbits immunized with recombinant protein on day 21 (Fig. 4b–e, *P* < 0.05, Mann–Whitney). Our results suggest that flagellin plasmid DNA immunization in rabbits is an effective vaccine strategy against *T. pallidum* dissemination and is significantly better than vaccination with the recombinant protein.

**Fig. 5:**
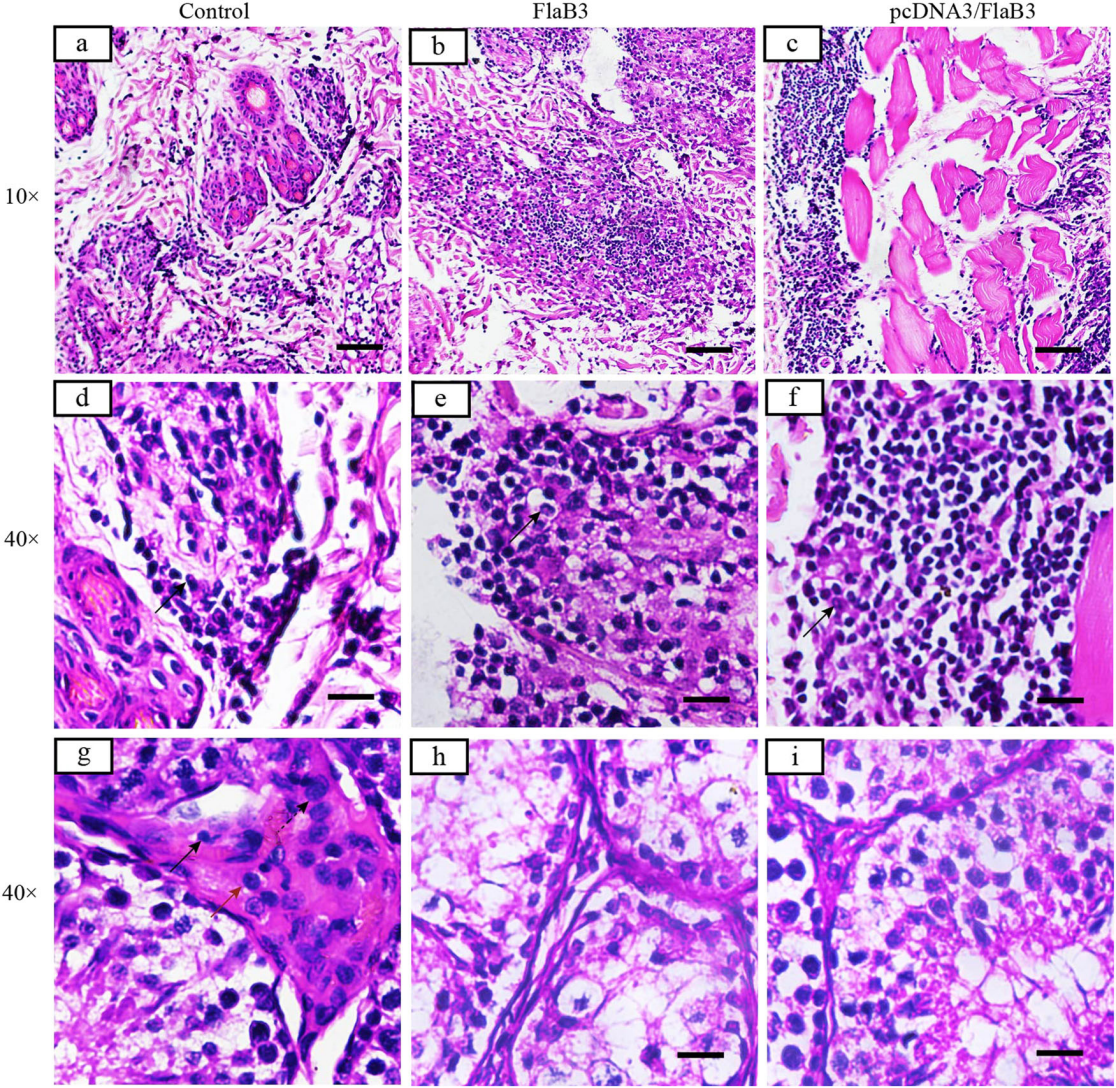
Histopathological changes in the skin and testicular tissues after *T. pallidum* infection in rabbits. Inflammatory infiltrates in pcDNA3/FlaB3- and FlaB3-immunized rabbits display a dense diffuse pattern (**b**, **c**) consisting predominantly of lymphocytes, macrophages, and plasma cells (solid arrow) (**e**, **f**). Interstitial inflammation of the testicular tissues is localized and predominantly lymphohistiocytic (solid arrow = lymphocyte; red arrow = macrophage; dashed arrow = plasma cell) in the control group (**g**) but not in the immunized rabbits (**h**, **i**). Skin and testicles of pcDNA3/FlaB3-, FlaB3-, and pcDNA3-immunized rabbits were sectioned and stained with H&E on day 21 after *T. pallidum* infection. Rabbits immunized with pcDNA3 were used as the controls (**a**)

### Immunization with pcDNA3/FlaB3 promotes cellular infiltration

H&E staining on day 21 postinfection was used to visualize the primary lesion sites and testicular tissue histology in rabbits infected with *T. pallidum*. Cellular infiltration of the skin (i.e., the area around the hair follicle filled with inflammatory cells, including lymphocytes, macrophages, and plasma cells) was significantly higher among the rabbits immunized with pcDNA3/FlaB3 than among the rabbits immunized with either recombinant flagellin or vaccinated with vector DNA alone (Fig. 5a–f). However, inflammation was also typically observed in the interstitial testicular tissue (i.e., the interstitial testicular tissue filled with inflammatory cells, including macrophages, plasma cells, and lymphocytes) in the rabbits vaccinated with vector DNA and PBS (Fig. 5j), but no inflammatory infiltrates were found in the interstitial testicular tissue of the rabbits immunized with either recombinant flagellin or pcDNA3/FlaB3 at day 21 postinoculation (Fig. 5h, i). The cellular infiltration in the testicle was in line with the qPCR results. Consistent with this observation, the pathogen burdens in the skin adjacent to the intradermal injection sites had the lowest treponemal burden in the rabbits immunized with pcDNA3/FlaB3, and many samples (spleen, liver, or testicle) contained low levels of treponemal DNA. Taken together, these results show that immunization of the rabbits with pcDNA3/FlaB3 or FlaB3 promotes cellular infiltration.

## Discussion

The results presented in this study showed that intramuscular immunization of New Zealand rabbits with the mammalian expression vector pcDNA3/FlaB3 elicited a high level of specific antibodies against flagellin and upregulated IFN-γ secretion. Furthermore, animals immunized with pcDNA3/FlaB3 displayed attenuated lesion development, inhibited *T. pallidum* dissemination to distant organ sites and promoted cellular infiltration. This high level of immunogenicity in New Zealand rabbits induced by DNA vaccination may have several explanations. The flagellar filament flagellin plays a major role in bacterial motility and elicits an innate immune response via recognition by TLR5, which contributes to activation of inflammatory responses and secretion of proinflammatory cytokines and chemokines during bacterial infections^18,34–36^. In addition, in our previous study, we found that *T. pallidum* flagellins could upregulate IL-6 and IL-8 via TLR5 and the MAPK/NF-ƙB signaling pathways^21^. In this study, increased IL-6 and IL-8 expression was observed in the in vivo-stimulated sera (Fig. S2). This cytokine production would appear to be critical for the development and enhancement of an initial adaptive immune response to flagellated *T. pallidum*. For full-fledged protection against *T. pallidum* infection, incorporation of the innate and adaptive immune systems is crucial. DNA vaccination has the potential to induce immunity to viral, bacterial, and tumor antigens and allergens^37,38^. Furthermore, DNA vaccines have demonstrated an adjuvant effect, resulting in elicitation of both humoral and cell-mediated immune responses and protection in a variety of animal models of infectious diseases^39,40^. However, to the best of our knowledge, the specific mechanisms involved in the expression, processing and presentation of flagellin antigen on APCs in New Zealand rabbits subsequent to plasmid DNA vaccination are unknown.

Anti-flagellin antibodies have been previously reported to play a role in passive protection against *T. pallidum*^11^. Overall, our results provided clear evidence that the recombinant flagellin produced only limited protection from *T. pallidum* infection in immunized New Zealand rabbits. However, this protective effect was significantly improved when the test rabbits were immunized with flagellin plasmid DNA prior to *T. pallidum* inoculation. The role of antibodies in anti-*T. pallidum* defense appears to be of minor importance, whereas long-lasting, protective immunity is believed to mainly rely upon Th1-type cell-mediated responses^33^. Previous studies demonstrated that antigen-specific IFN-γ was important for Th1-type immune responses and that Th1 cytokine-mediated macrophage activation promoted phagocytosis of opsonized *T. pallidum*^33,41–45^. Other studies have suggested that activated CD8+ cells participate in local syphilis clearance^32^. Therefore, the macrophage-activating effectors IFN-γ and CD8+ cells were assayed. The results show overproduction in the DNA-vaccinated New Zealand rabbits compared to that in the recombinant protein-vaccinated rabbits. The DNA-vaccinated rabbits showed upregulation of IFN-γ and CD8+ cells 21 days after immunization specifically in response to the presence of flagellin stimulation. This response resulted in low treponemal burdens for New Zealand rabbits infected intradermally with *T. pallidum*. These results strengthen the conclusion that a protective immune response in our case has been specifically elicited in the New Zealand rabbits by plasmid DNA encoding the flagellin gene.

Phagocytosis of *T. pallidum* by cytokine-activated macrophages aids in bacterial clearance and lesion resolution during early syphilis^42^. During syphilis infection, primary lesions in humans and primary and secondary lesions in rabbits contain predominantly CD4^+^ T cells, macrophages, NK cells, and plasma cells^41,46^. *T. pallidum* is a highly invasive pathogen that is capable of penetrating through abrasions in the skin or genital mucosa, invading the tissue barrier and gaining rapid entry to the bloodstream^3^. The characterization of early syphilis infection is a broad immune inflammatory response in the skin and mucosa^6^. As a highly evolutionarily conserved PAMP (pathogen-associated molecular pattern), flagellin can be recognized by the cell surface through TLR5 and the cytosolic NOD-like receptor protein 4 (NLRC4) inflammasome receptor NAIP5, which contributes to activation of inflammatory responses and secretion of proinflammatory cytokines and chemokines during bacterial infections^35,47,48^. The *T. pallidum* flagellin induced MMP-9 and MMP-13 expression in keratinocytes, which are involved in activation and regulation through the NF-κB and MAPK signaling pathways^22^. These results suggest that the *T. pallidum* flagellin may be crucial for triggering inflammation, which may contribute to skin inflammatory responses and keratinocyte-mediated ECM degradation during *T. pallidum* infection. Hence, the ability of FlaB3 to stimulate nonspecific host resistance may be relevant for anti-*T. pallidum* protection. In particular, inflammatory cytokines induced by the plasmid encoding the FlaB3 gene may significantly contribute to protection due to their well-known ability to activate host phagocytes for efficient control of bacterial invasivity^36^. Histological analysis of the primary lesion sites in the control and immunized animals revealed a significant increase in cellular infiltration in the DNA-vaccinated rabbits at day 21 postinfection. Strikingly, abundant lymphocytes, macrophages and plasma cells were discovered within the primary lesion sites. Significant inflammatory responses were observed in the rabbits vaccinated with vector DNA alone but never in the testicular tissues of the rabbits immunized with pcDNA3/FlaB3 and recombinant flagellin. In line with the trend observed by darkfield microscopy, the primary lesions from the immunized animals had few treponemes present per field, and these organisms were nonmotile. The results of our study also provide evidence that the FlaB3-specific antibodies produced by these immune cells facilitate *T. pallidum* retention at the primary lesion sites by inhibiting treponemal dissemination. In particular, this protective effect was more pronounced in the New Zealand rabbits immunized with pcDNA3/FlaB3 than in the rabbits immunized with recombinant flagellin. However, these histological analyses were performed at a single time point and therefore did not represent the dynamic properties of cellular infiltration.

The key component in *T. pallidum* pathogenesis is vascular dissemination, and therefore effective vaccination needs to target treponemes within the vasculature to inhibit *T. pallidum* seeding into secondary sites^9^. Analysis of the treponemal burden in the blood, liver, spleen, and testicles using qPCR revealed that pcDNA3/FlaB3 immunization inhibited *T. pallidum* invasion into distant tissue sites. In particular, the most significant decrease in the *T. pallidum* DNA concentration was observed in the blood, spleen, or testicles between the immunized and control animals. Previous investigations have confirmed that Tp0751 is capable of mediating spirochete attachment to endothelial cells when heterologously expressed by a noninfectious *Borrelia burgdorferi* strain^49^. Moreover, Lithgow et al. reported that animals immunized with Tp0751 displayed attenuated lesion development, inhibition of *T. pallidum* dissemination and increased cellular infiltration at lesion sites^10^. Our work reported here used a recombinant flagellin to stimulate augmented production of MMPs which are involved in the degradation of various components of the extracellular matrix (ECM) to facilitate cell migration and invasion^22^. Therefore, the qPCR results clearly demonstrate that pcDNA3/FlaB3 is a promising vaccine candidate, because immunization with this protein significantly reduces *T. pallidum* dissemination within the host. However, these results must be interpreted with caution because the qPCR is capable of detecting DNA from both live and dead organisms.

Notably, the statistical data in this study are not generally representative due to the small sample size and the outbred nature of the rabbits used as the animal model. However, the findings from this study can be used to guide experiments in the future as a parameter to determine an adequate sample size. Some *T. pallidum-*elicited infections may convert from an initial acute infection to a form of long-term latent infection by this microbe^50^. If the bacterial loads present in the liver, spleen, blood, or testicles in this study are not be removed completely following initial infection, the limited survival of *T. pallidum* in the organs will be a negative factor. Thus, further investigation is needed to evaluate the relative antimicrobial activity among immunized rabbits with different genetic backgrounds.

## Materials and methods

### Plasmid and bacterial strains

The pET28a/FlaB3 plasmid encoding the flagellin gene of *T. pallidum* was constructed to express the recombinant flagellin protein in *E. coli* BL21 (DE3); the protein was expressed and purified as described in a previous report^12^. The mammalian expression vector pcDNA3/FlaB3 was constructed from pET28a/FlaB3 using appropriate restriction sites flanking the *flaB3* gene. The *T. pallidum* subsp. *pallidum*, Nichols strain was propagated by intratesticular inoculation of adult New Zealand White rabbits as previously described^51,52^. All animal experiments were approved by the Animal Welfare Committee of the University of South China and conducted in accordance with the institutional regulations.

### Expression of the *flaB3* gene in transfected cells

HeLa 229 cells (ATCC, CCL-2.1, USA) were transfected with either pcDNA3 or pcDNA3/FlaB3 using the Lipofectamine^TM^ 2000 Reagent (Invitrogen, Carlsbad, CA, USA) according to the manufacturer’s instructions. Transfected HeLa cells were harvested and detected using immunoblotting analysis with antibodies against FlaB3. The primary anti-FlaB3 antibodies were provided by the Institute of Pathogen Biology at the University of South China. The secondary antibody was horseradish peroxidase-conjugated secondary goat anti-rabbit IgG (GeneTex, San Antonio, TX, USA). Finally, the ECL Prime Western Blotting Detection Reagent (Thermo Fisher Scientific, Fremont, CA, USA) was used as recommended to visualize the protein bands with a G:BOX Chemi XX9 (Syngene, Cambridge, UK) digital imager.

### Immunization of the rabbits

We used a cohort of eight male SPF New Zealand White rabbits (3.0 kg, 13–15 weeks of age, the Animal Department of the University of South China) with negative VDRL and FTA-Ab serology. Two experimental groups of rabbits with three animals per group were injected intramuscularly with either purified recombinant flagellin (150 μg) emulsified in complete Freund’s adjuvant (Sigma, MO, USA) or pcDNA3/FlaB3 (250 μg) in PBS. Two animals randomly selected from the cohort of eight rabbits were injected intramuscularly with pcDNA3 (150 μg) in PBS. The experimental group rabbit were immunized three times at 2-week intervals. As a control, the data derived from the rabbits injected with PBS gave the same result as the pcDNA3 control group and therefore were not shown in this report. Serum samples were collected by ear vein bleeding 14 days after the commencement of immunization.

### Challenge procedure

At 21 days after the final immunization and 10 days after the control animals were bled, the rabbits’ backs were shaved, cleansed with 70% ethanol and injected intradermally with 0.1 ml of 1 × 10^7^*T. pallidum* subsp*. pallidum* (Nichols strain) per ml in 0.9% saline in each of eight spots^10,53^. The challenge sites were monitored daily for erythema, induration, and ulcerative lesions and measured every 3 days to assess the lesion diameters. Aspirates were taken from the lesions 21 days post-challenge and examined by darkfield (DF) microscopy (Nikon Canada, Mississauga, ON, Canada) for the presence of treponemes.

### Measurement of serum antibody responses in the rabbit

A small volume (1 ml) of blood was collected from the rabbit ear veins, followed by centrifugation for serum separation. The serum samples were tested for specific antibodies using the indirect ELISA method. Purified recombinant flagellin (pET28a/FlaB3) was used as the coating antigen. Horseradish peroxidase-conjugated secondary goat anti-rabbit IgG (1:1000) was used as the secondary antibody. ELISA was performed to examine the A value at the 450 nm wavelength (BioTek Instruments, Winooski, VT, USA). Each experiment was repeated three times.

### Evolution of cellular responses

The induction of IFN-γ/CD8+ cells was evaluated using a rabbit interferon-γ (IFN-γ) ELISA kit (Cusabio Biotech Co., Ltd., Wuhan, China) and a rabbit cluster of differentiation 8 (CD8) ELISA kit (BlueGene Biotech Co., Ltd., Shanghai, China) according to the manufacturers’ instructions. The quantity of IFN-γ was reported as pg/ml and CD8+ cells was reported as ng/ml according to the standard curves.

### Cytokine assays

The IL-6 and IL-8 concentrations in sera from the immunized rabbits were determined using a specific enzyme-linked immunosorbent assay (ELISA) kit from Cloud Clone Inc. (USA) according to the manufacturer’s instructions. The quantity of each secreted cytokine was reported as pg/ml according to the standard curve.

### Extraction and purification of *T. pallidum* DNA from tissues

Genomic DNA (gDNA) was extracted from tissues collected from *T. pallidum*-challenged rabbits using a method adapted from the Qiagen QIAamp DNA Mini Kit (Qiagen, Shanghai, China). Lysis Buffer (180 ml; 10 mM Tris, pH 8.0, 0.1 M EDTA, and 0.5% SDS) was added to ~25 mg of tissue (~10 mg for spleen) and homogenized using the Qiagen TissueLyser LT operating for 40 s at 30 Hz. Proteinase K (5 mg) was added, and the digestion was performed overnight at 56 °C. Then, the samples were treated with RNase A (400 µg), followed by incubation at 70 °C for 10 min. Three tissue samples from each rabbit organ were analyzed for reproducibility. The tissue extracts were eluted in 100 µl of Gibco PCR Grade Distilled Water (Thermo Fisher Scientific) and stored at −80 °C. DNA was quantified using a Beckman Coulter DU 730 Life Science UV/Vis Spectrophotometer (Beckman Coulter Canada, Mississauga, ON, Canada).

### Quantitative PCR

Quantitative real-time PCR was performed on gDNA extracted from the *T. pallidum*-challenged rabbit tissues using a SYBR green I assay. The *T. pallidum* gDNA was quantified using primers targeting a 285-bp region of the endoflagellar sheath protein (*flaA*) gene (GenBank accession number M63142). The sense primer (5′-AACGCAAACGCAATGATAAA-3′) annealed to bases 475 to 494 and the antisense primer (5′-CCAGGAGTCGAACAGGAGATAC-3′) annealed to bases 738 to 759 of *flaA*. The rabbit gDNA was quantified with primers targeting a 267-bp region of exon 1 of the collagenase-1 precursor (*MMP-1*) gene (GenBank accession number M17820). The sense primer (5′-TTGCTTCTTCACACCAGAATGCTGT-3′) annealed to bases 300 to 324 and the antisense primer (5′-GCGTGATCAGGCACTATGTAGCAAT-3′) annealed to bases 542 to 566^10^. All primers were ordered from Integrated DNA Technologies (Sangon Biotech, Shanghai, China). The quantitative real-time PCR reactions were performed in 20-μl reaction volumes according to the manufacturer’s instructions (Qiagen, Shanghai, China). The amount of gDNA isolated was variable between tissue types but was normalized within each tissue type based on the lowest gDNA concentration obtained from the spectrophotometric measurements. A standard curve was created for *flaA* using a 10-fold serial dilution from 10^7^ to 10^1^ copies of linearized plasmid DNA with an efficiency of 99.2% and an R^2^ value of 0.990. A standard curve was created for *MMP-1* using a twofold serial dilution of rabbit gDNA from 100 to 0.0488 ng μl^−1^ with an efficiency of 99.1% and an R^2^ value of 0.995. The assays were run on the LightCycle 96 apparatus (Roche, Basel, Switzerland). The PCR conditions for *flaA* were as follows: 95 °C for 8 min, followed by 40 cycles of 95 °C for 15 s, 56 °C for 20 s, and 72 °C for 30 s, an additional extension step at 84 °C for 10 s, and a final denaturation step of 15 s at 95 °C; additionally, a melting curve was performed from 60 to 95 °C over 20 min. The PCR conditions for *MMP-1* were as follows: 95 °C for 10 min, followed by 40 cycles of 95 °C for 15 s, 55 °C for 20 s, and 72 °C for 40 s, and a final denaturation step of 15 s at 95 °C; a melting curve analysis was performed from 60 to 95 °C over 20 min. Each assay was run with the following four controls: (1) no template control; (2) no amplification control (no Taq polymerase); (3) no primer control; and (4) positive control with a known concentration or copy number of rabbit gDNA or linearized flaA plasmid DNA, respectively.

### Histology

Biopsy punch samples (2 mm) were taken from a single lesion and testicle from each rabbit at day 21 postinfection and sent to the University of South China. The samples were fixed in formalin, mounted, bisected, and vertically sectioned with a 4-mm thickness. The samples were processed with hematoxylin and eosin staining for microscopic analysis to determine the abundance of immune cells, including lymphocytes, macrophages, and plasma cells.

### Statistical analysis

The data were analyzed using Student’s *t*-tests and nonlinear regression with an extra sum of squares F-test to investigate any apparent differences between the test and control groups. Differences were considered significant at the *P* < 0.05 level. The GraphPad Prism 5.0 software (San Diego, CA, USA) was used for statistical analysis of the data.

## Electronic supplementary material

Fig S1

Fig S2

Fig S3
