# Correction

**DOI:** 10.1080/22221751.2019.1583955

**Published:** 2019-03-08

**Article title:** Immunogenicity and protective efficacy against Treponema pallidum in New Zealand rabbits immunized with plasmid DNA encoding flagellin

**Authors:** Zheng, K., Xu, M., Xiao, Y., Luo, H., Xie, Y., Yu, J., Tan, M., Li, Y., Zhao, F., Zeng, T., & Wu, Y.

**Journal:***Emerging Microbes & Infections*

**Bibliometrics:** Volume 7 (2018)

**DOI:**https://doi.org/10.1038/s41426-018-0176-0

After this article was published online, the authors brought to our attention that they did not acknowledge that the methods drew heavily on the work previously published by Lithgow et al. (please see the reference below).

The authors wish to clarify that they followed the experimental method outlined by Lithgow et al. (including the regents referenced) but with different experimental conditions.

Parts of the Methods section (principally “Challenge procedure”, ”Extraction and purification of T. pallidum DNA from tissues”, “Quantitative PCR” and “Histology”) overlap with Lithgow et al.

The authors apologize to Lithgow et al. and to readers for this oversight.

Reference

Lithgow KV, Hof R, Wetherell C, Phillips D, Houston S, Cameron CE. A defined syphilis vaccine candidate inhibits dissemination of Treponema pallidum subspecies pallidum. Nat Commun. 2017;8:14273. doi:10.1038/ncomms14273.

