# Supplementary figures and images for: Immunogenicity and protective efficacy against Treponema pallidum in New Zealand rabbits immunized with plasmid DNA encoding flagellin
Source: Emerg Microbes Infect. 2018 Nov 7;7:177. doi: 10.1038/s41426-018-0176-0 (PMC6220273; doi:10.1038/s41426-018-0176-0)

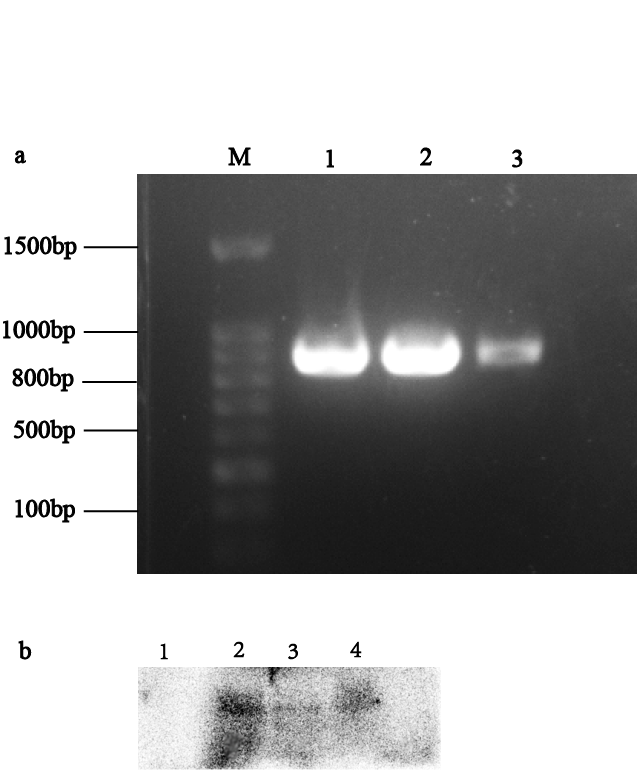

Supplement: Supplementary file 1 — Fig S1 [file 41426_2018_176_MOESM1_ESM.tif]

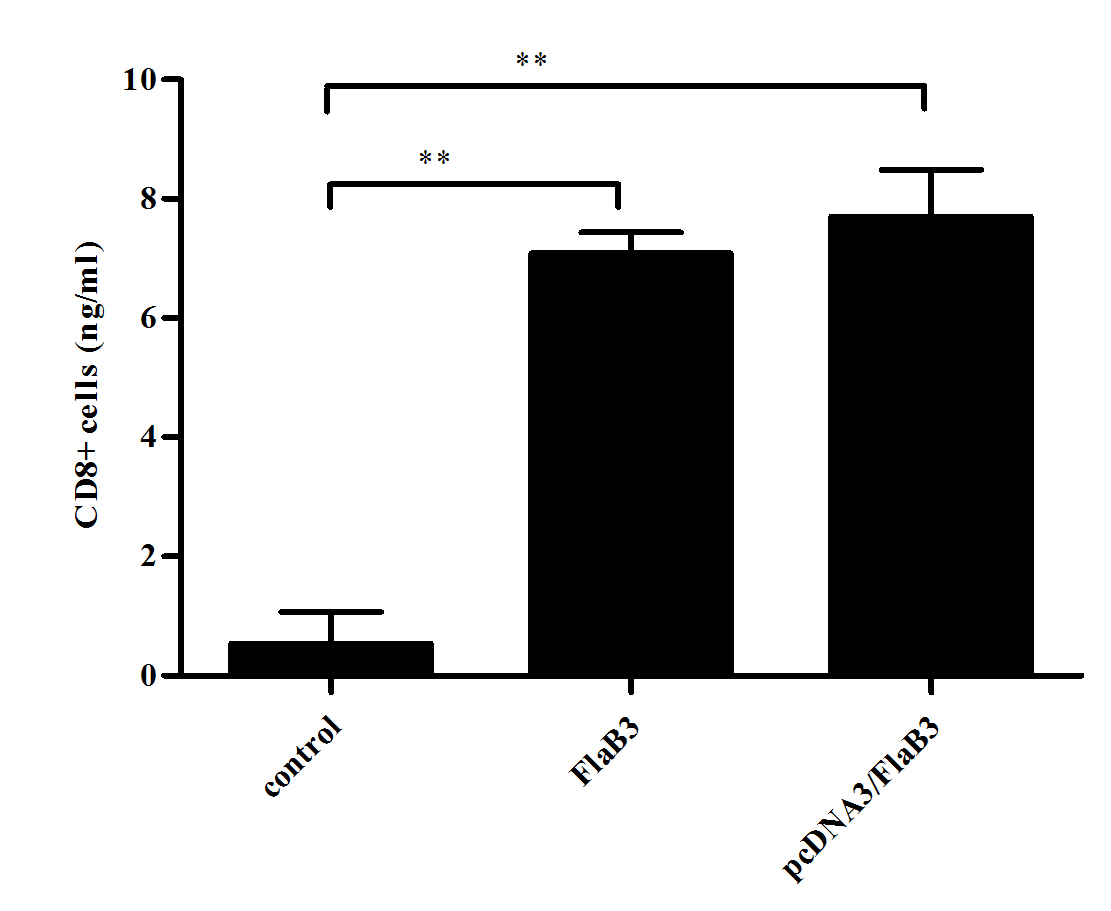

Supplement: Supplementary file 2 — Fig S2 [file 41426_2018_176_MOESM2_ESM.jpg]

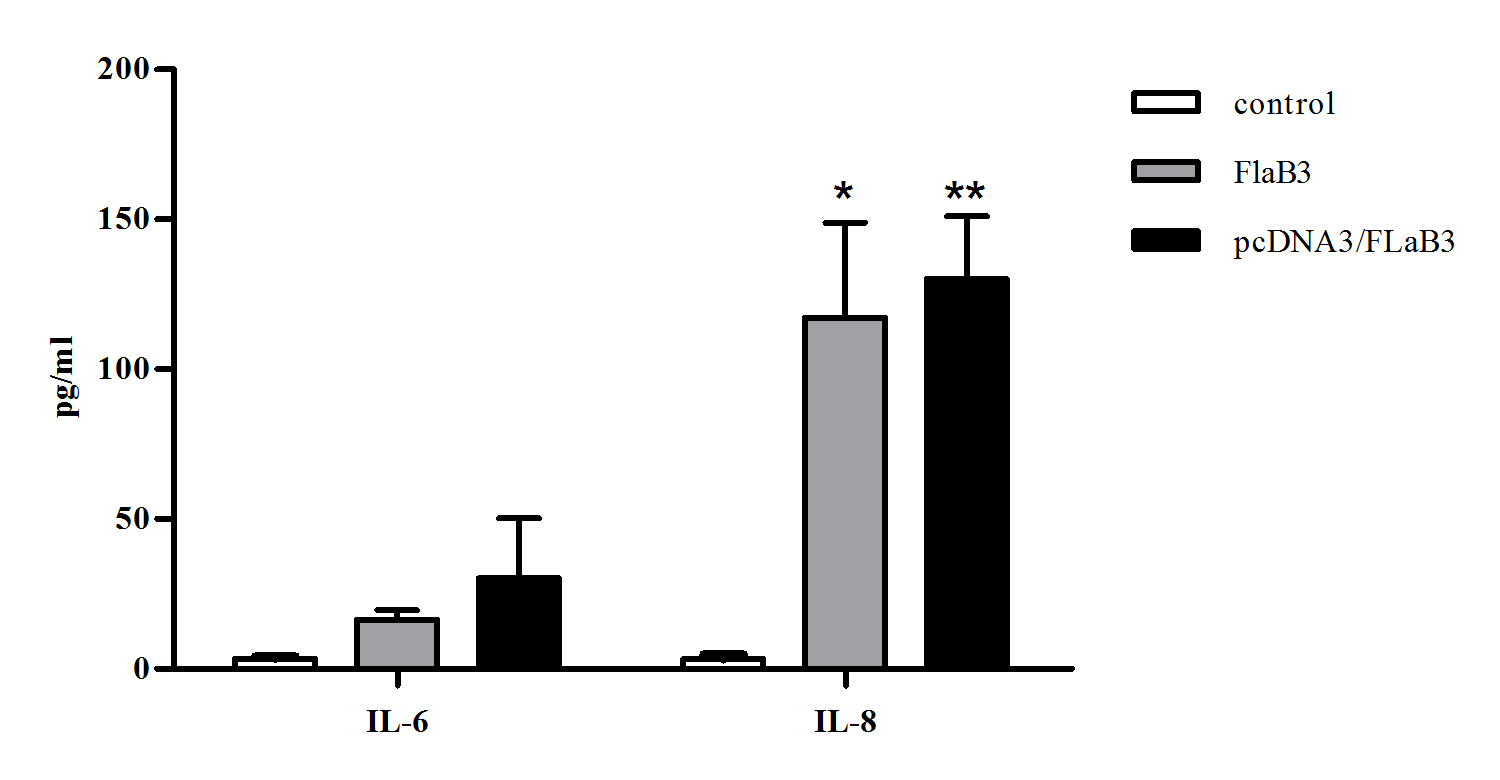

Supplement: Supplementary file 3 — Fig S3 [file 41426_2018_176_MOESM3_ESM.jpg]
